# Reexamining the Role of Amyloid β Clearance from the Brain: Exporting Labile Iron from the Interstitial Fluid Performs a Protective Function

**DOI:** 10.3390/ijms27031485

**Published:** 2026-02-02

**Authors:** Steven M. LeVine

**Affiliations:** Department of Cell Biology and Physiology, University of Kansas Medical Center, 3901 Rainbow Blvd., Mail Stop 3043, Kansas City, KS 66160, USA; slevine@kumc.edu

**Keywords:** Alzheimer’s disease, amyloid β, amyloid precursor protein, anemia of chronic disease, ferroportin, inflammation, infection, iron, iron regulatory elements, labile iron, LDL receptor-related protein 1, proinflammatory cytokines, siderophore

## Abstract

Advantageous functions have been attributed to amyloid β, which helps explain its expression despite a propensity to aggregate. Besides supporting cognitive processes, it has antimicrobial activity, e.g., amyloid β can entrap pathogens or disrupt their membranes. Since iron is an essential element for invading organisms, limiting its availability is an antimicrobial strategy. This can be achieved by various means, such as reducing circulating iron, as is the case for anemia of inflammation or anemia of chronic disease, which may occur in Alzheimer’s disease. The protein lactoferrin both sequesters iron and generates proteolytic fragments with antimicrobial properties, and amyloid β may have similar traits. Amyloid β, which is derived from proteolytic cleavage of amyloid precursor protein, directly inhibits microorganisms. In addition, it binds redox-active metals, such as iron and copper. After being generated, amyloid β can enter the interstitial fluid and undergo clearance by a variety of mechanisms (e.g., glymphatic system, transport across the blood–brain barrier, and uptake by microglia or astrocytes). This clearance, together with its small size and iron-binding properties, positions amyloid β to perform a surveillance function to access, capture, and export labile iron. By removing extraneous iron, amyloid β also helps to limit metal-catalyzed reactions that cause tissue damage. In summary, besides preventing the aggregation and neurotoxicity of amyloid β, the clearance of amyloid β from the CNS may serve a surveillance function to remove loosely bound iron to avert injury by redox reactions and enable amyloid β to function as a mammalian siderophore making iron unavailable to invading microorganisms.

## 1. Introduction

Amyloid precursor protein (APP) is evolutionarily conserved with homologues identified from invertebrates to vertebrates [1,2,3]. One of its proteolytic processing products, amyloid β, has a common sequence between humans and numerous vertebrates [3], and it has been implicated to have a critical role in the pathophysiology of Alzheimer’s disease rather than just being a byproduct of disease activity [4]. In contrast to its role in disease, various normal functions have been attributed to APP and its proteolytically cleaved fragments. These include, but are not limited to, contributing to neurodevelopment, synaptic plasticity, cell adhesion, metabolism, mitochondrial function, protection against neuronal stress and injury, angiogenesis, etc. [5,6,7,8,9,10]. Additionally, it has been ascribed a role in the homeostasis of metals within the brain [11,12].

Redox-active metals, such as iron and copper, catalyze essential biochemical reactions within cells, such as those within the mitochondrial electron transport chain. The brain has extra requirements for metal-catalyzed reactions, e.g., neurotransmitter synthesis and in support of myelin formation. However, redox-active metals have the potential to catalyze reactions that have damaging effects, e.g., the formation of reactive oxygen and reactive nitrogen species [13,14,15,16,17]. Given the combination of a high requirement for metals and their potential for tissue damage, the brain utilizes intricate mechanisms to control their availability and safe handling. A major component of this process is the blood–brain barrier, which regulates the transit of metals and other molecules into and out of the central nervous system (CNS). Other reviews have discussed the various proteins and mechanisms involved in the transport and metabolism of iron and copper within the healthy brain and in the context of Alzheimer’s disease [18,19,20,21,22]. A less well-known participant in this process is the proteolytically cleaved fragment of APP, i.e., amyloid β.

APP has been connected to the export of iron from cells in the CNS, but the role of amyloid β in this process has not been adequately explored. In this review, we will assess the findings of previous studies and make the case that amyloid β is an active participant in the export of iron from the CNS under normal circumstances. We will examine how APP and its proteolytic fragments help manage iron homeostasis and address how this functions as a defense mechanism to prevent both tissue injury and infections in the CNS.

## 2. APP Transcript and Iron Regulatory Elements

Iron regulatory elements (IREs) are located in the 5′ or 3′ untranslated regions (UTRs) of various mRNAs whose expression is tied to iron metabolism. Iron regulatory proteins (IRPs) bind the IREs to modulate translation. The 5′ UTR of APP has an IRE that is responsive to IRP-1 and shares homology with the IRE in the mRNA transcript for the iron storage protein ferritin [23,24]. Besides iron, the UTR downstream of the IRE can respond to IL-1 (IL-1 responsive acute box element) to increase the translation of APP, as well as the translation of ferritin [24,25,26,27,28].

Levels of iron influence the expression of APP and its proteolytic products. In ARPE-19 cells, a cultured retinal pigment epithelial cell line, the translation of APP increased, as did the levels of its proteolytically cleaved products (Aβ42, C83, and C99), after iron levels were elevated [29]. In SHSY5Y cells, a neuroblastoma cell line, the addition of ferric ammonium citrate increased production of APP, β-secretase activity, and Aβ42 levels [30]. In BV-2 cells, a microglial cell line, ferric chloride increased both APP and Aβ42 levels [31]). In HEK 293 cells, human embryonic kidney cells, transfected with APP, moderate levels of iron (via hemin) increased levels of soluble APP, but not proteolytic fragments [32]. In cortical neurons, prepared from rat embryos, treatment with ferric ammonium citrate caused no change in full-length APP, but increased the levels of the carboxy-terminal fragment α. Furthermore, the secreted form of soluble APPα decreased but the intracellular levels of soluble APPα increased, while levels of the secreted forms of both soluble APPβ and soluble Aβ40 decreased [33]. In normal mice, restricting iron via chelation reduced iron content in the brain and lowered APP and secretase enzymes levels [34]. Together, these mixed results illustrate that the production of APP and its proteolytic fragments, such as amyloid β, can be responsive to the concentration of iron, but the results are dependent on the specific experimental condition.

Mechanistically, an increase in the cellular ferrous iron level is thought to release IRP-1 from the IRE in the APP 5′-UTR [35]. Then, eIF4F, a protein complex involved in translation initiation, binds to the IRE and recruits ribosomes to promote APP translation [35,36,37]. Furthermore, iron may increase the activity of secretases to produce proteolytically cleaved products [30,32]. Together, these results suggest that elevated cellular iron levels increase the production of APP and its cleavage products, while during low iron conditions, IRP-1 remains bound to the APP IRE to repress APP translation [35].

The regulation of APP translation by iron has similarities to that for ferritin [24,35]. However, the regulation of APP translation by iron may not be simple and likely involves additional regulations; a microRNA miR-346 also binds the 5′-UTR of APP overlapping with the IRE and the IL-1 responsive acute box element [38]. In conjunction with argonaute 2, miR-346 has been suggested to displace IRP-1, and functions to increase APP translation when iron levels are decreased, e.g., during chelation in primary human neuronal enriched cultures [38]. Additionally, poly(C)-binding proteins, which have a variety of functions including being chaperones of iron and regulating the processing of RNA [39], have been suggested to interact with the acute box element in the 5′-UTR of APP to help regulate its translation in response to IL-1 in the presence of iron [27]. This function is in line with the role of poly(C)-binding proteins in mRNA regulation in response to iron conditions as well as the various regulatory mechanisms for APP mRNA [39,40]. Thus, there may be multiple mechanisms by which the excess or deficiency of iron affects APP translation and these may be dependent on the cell type, disease state, or other cellular or environmental factors.

## 3. Cellular Iron Export in the Brain—APP and Ferroportin

Ferroportin is expressed by neurons and oligodendrocytes [41]. Ferroportin is a transmembrane protein that functions as an antiporter; two protons are coupled with the exchange of one ferrous iron, resulting in the export iron from the cytoplasm to the surface of the cellular membrane [42]. Soluble APP was found to co-precipitate with ferroportin and promoted the export of iron [43]. Primary neurons that are deficient in APP retain more iron than wild-type neurons [43], and when other neurodegenerative conditions are present (i.e., tau deficiency or the Huntington’s disease mutation), a mistrafficking or reduction of APP resulted in the accumulation of iron in primary cortical neurons or in the brain, indicating a role of APP in iron export [44,45]. Given the propensity of APP to regulate the iron exporter ferroportin, Belaidi et al. [46] proposed that APP may function to counteract the age-associated increase of iron within the brain.

A specific domain within APP is thought to stabilize ferroportin in the plasma membrane, thereby increasing the efflux of iron into the interstitial space of the brain [47], but there is a requirement to convert the exported ferrous iron to the ferric state before its removal or transport by transferrin. Ceruloplasmin acts as a ferroxidase that converts ferrous iron to ferric iron and is thought to have a key role in the efflux of iron [48]. Although ceruloplasmin, functioning with a GPI anchor, is produced by astrocytes, it is also synthesized by the choroid plexus that produces a secreted form which could enable its more widespread use in the CNS [48,49,50,51,52]. Thus, ceruloplasmin may function with neuronal ferroportin [53].

If ceruloplasmin is deficient, then iron accumulates in various regions of the CNS (e.g., brainstem, cerebellum, and retina), which supports the notion that it has a role in the export of iron from the brain [54]. In contrast to this model, hephaestin was found to be key to mediating iron efflux; in cultured hippocampal neurons, when the expression of hephaestin was decreased, neurons accumulated iron and its export was reduced, while decreasing the expression of APP did not alter iron efflux [55]. Hephaestin is thought to complex with ferroportin, i.e., they form a FRET pair in cultured HEK293T cells, but this interaction was not observed between hephaestin and full-length APP [56]. However, soluble APP increased the occupancy of ferroportin in the membrane and it appeared to promote iron efflux [47,56]. Thus, ferroportin likely functions together with soluble APP, ceruloplasmin, and/or hephaestin. However, another protein, hepcidin, causes the down-regulation of ferroportin as well as other proteins involved with iron transport [57,58,59] adding additional layers of influence on the export of iron by ferroportin and its associated proteins. Activation of the hepcidin/ferroportin axis is thought to help control infections [60] and may be increased in the aged brain [61].

## 4. Iron Binding Amyloid β

APP located at the plasma membrane can be digested by β secretase and then by γ secretase to produce amyloid β, which can then undergo various fates. For example, it can remain associated with the plasma membrane, bind to GM1 ganglioside in lipid rafts, or enter the extracellular space where it can bind ApoE and undergo endocytosis via low-density lipoprotein (LDL) receptor-related protein 1 (LRP-1) [62]. Interestingly, overexpression of the carboxyl-terminal fragment of APP, which includes amyloid β, lowered iron and copper levels in the mouse brain [63], suggesting a role for the amino acid sequence of amyloid β in iron export.

Numerous studies have demonstrated that iron binds amyloid β in vitro [64,65,66,67]. Other studies have shown that iron also binds to amyloid in vivo; iron is bound to plaques in CNS tissue from both patients with Alzheimer’s disease and its animal model [68,69,70,71].

There are multiple residues on amyloid β that are thought to bind iron, e.g., several histidine residues and possibly glutamic acid and aspartic acid [65,67]. Amyloid β does not require an oxidase in order to bind iron, since it can bind both ferrous and ferric iron [64,65,66,67,72]. Amyloid β has an affinity for ferric iron (K_d_ = 6.3 × 10^−21^) that is similar to that of transferrin and has a lower affinity of ferrous iron (K_d_ = 5.0 × 10^−12^) [72]. Some studies suggest that the ferric iron bound to amyloid β can be reduced to ferrous iron [66], or that the iron bound to amyloid plaques is redox-active [73]; however, the redox activity of iron bound to amyloid β is uncertain [74]. Besides iron, heme has been found bound to amyloid β and it can catalyze damaging oxidative reactions [13,16,75,76].

The ability of amyloid β to bind ferrous iron [64,65,72] differs from the binding of iron to transferrin which needs an oxidase, such as ceruloplasmin or hephaestin, to convert ferrous iron to the ferric form before binding. Besides not needing an oxidase to facilitate binding, the efficiency of amyloid β binding to iron is further enhanced by the following multiple factors: it has multiple sites for binding with iron (vs. two for transferrin), the affinity of iron is relatively high (e.g., it is comparable to that for ferric iron and transferrin), and it is small in comparison to other transport proteins (e.g., ~4.3–4.5 kDa for amyloid β vs. ~76.5–79.6 kDa for transferrin) [65,67,72,77]. Being small favors enhanced iron binding by multiple ways as follows: amyloid β has less steric hindrance than larger molecules; it potentially has more flexibility; it has a greater surface area to bind iron relative to its volume; and it can access molecular spaces which are inaccessible to larger molecules.

## 5. Clearance of Amyloid β from the Brain

Amyloid β is cleared from the brain in multiple ways [78]. Both soluble and fibrillary forms of amyloid β get taken up by microglia [79] with the fibrillary form removed via phagocytosis and the soluble form removed by macropinocytosis, which is dependent on a pseudopod driven by actin to form a phagosome, with delivery to the lysosome for subsequent proteolytic degradation [80,81,82,83]. Besides microglia, astrocytes and neurons can also take up amyloid β by macropinocytosis, albeit with lower efficiency than microglia [82]. In the presence of ApoE, astrocytes take up amyloid β by the LDL receptor(s) and process it for degradation [84]. ApoE also expedites amyloid β proteolytic degradation within the interstitial fluid and microglia [85]. Amyloid β is proteolytically degraded by a variety of proteases, such as insulin-degrading enzyme, matrix metallopeptidases, neprilysin, endothelin-converting enzyme, and others [78,86]. Besides degradation via the lysosome within cells, astrocytes also secrete various proteases that can digest amyloid β [78,87].

Amyloid β can also be cleared by an LRP-1 mediated mechanism on endothelial cells of brain capillaries, wherein it undergoes transcytosis to the peripheral circulation [79,88,89,90]. This is likely an important route for amyloid β clearance from the brain [91]. LRP-1 is also a receptor of ApoE, which may facilitate amyloid β clearance across the blood–brain barrier with the help of P-glycoprotein, particularly when amyloid β is bound to ApoE2, ApoE3, or α2-macroglobulin, but not ApoE4 [92,93]. Other than transcytosis across the brain endothelial cells [90], amyloid β may also be cleared by smooth muscle, fibroblasts, neurons, or choroid plexus epithelial cells [94,95,96,97]. Interestingly, iron induced the expression of LRP-1 and ApoE within cultured neurons, and increased the transcriptional and translational expression of ApoE within cultured astrocytes [98], suggesting a positive feedback mechanism.

In addition to LRP-1, the efflux of amyloid β likely involves other functionally associated proteins such as the ATP-binding cassette (ABC) transporter ABCB1 (also known as P-glycoprotein) and phosphatidylinositol-binding clathrin assembly protein [99,100]. It is possible that the LRP-1/ApoE/amyloid β pathway may function more at the blood–cerebrospinal fluid barrier at the choroid plexus [94], with less efficiency at the blood–brain barrier [101]; therefore, other pathways may be involved in moving amyloid β from the brain to the blood, such as LRP-2, very LDL receptor, P-glycoprotein, other ABC transporters, etc. [86,102].

The glymphatic system, which includes the drainage of interstitial fluid along the paravenous route, is thought to help clear amyloid β [103]. Aquaporin-4, a water channel expressed by astrocytes, is a key contributor to the glymphatic system, and perturbation of its function leads to reduced clearance of amyloid β from the CNS [104,105].

## 6. Is Clearance of Labile Iron in the Interstitial Fluid a Function of Amyloid β?

Given that amyloid β has been associated with the development of Alzheimer’s disease and cerebral amyloid angiopathy, its clearance from the brain has been understood to protect neurons from amyloid β-mediated pathology [78]. It is tempting to speculate that amyloid β is an example of antagonistic pleiotropy [106,107]. For example, amyloid β would have advantageous functions in relatively younger individuals while negative consequences of amyloid β, e.g., elevated levels of toxic aggregates, would occur long after reproduction, i.e., after most forms of parental investment have ceased. Furthermore, studies suggest that older individuals, e.g., postmenopausal, can contribute to species survival [108,109]. Thus, by this model, natural selection has delayed the onset of amyloid β pathogenicity until very late in life, past what would have been grandparenting years for our ancestors; after this point, there is little selective pressure to further delay its pathogenic effects.

Numerous native functions for APP and amyloid β have been put forth, many of which facilitate the development and function of the nervous system [110]. Primates have evolved multiple mechanisms and exert considerable effort to clear amyloid β from the brain [78], rather than simply preventing its production and/or eliminating extracellular export in the first place. It is possible, then, that this clearance itself *is* the advantageous function. In other words, the clearance of amyloid β is not solely to prevent its pathological effects but is instead an adaptive function. What is this function? Here, we propose that the clearance of amyloid β is an adaptive function to export iron from the brain in order to protect it from infection and oxidative tissue damage.

**Figure 1:**
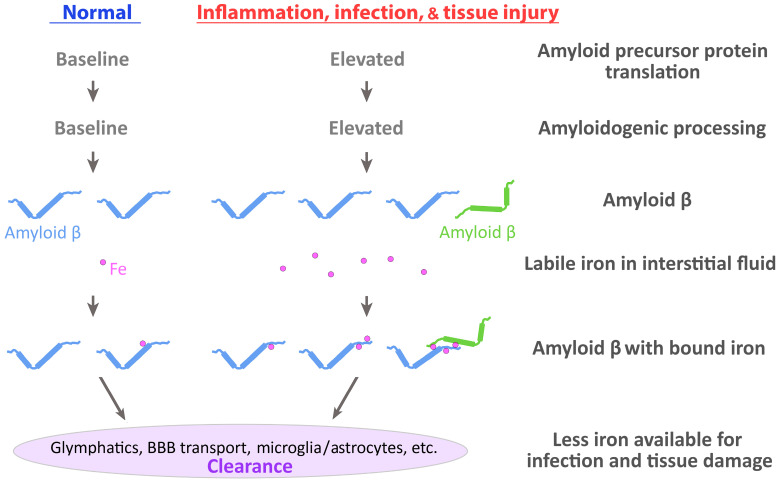
**The clearance of labile iron from the interstitial fluid by amyloid β.** During inflammation, infection, or tissue injury, the translation and amyloidogenic processing of amyloid precursor protein increases above baseline levels. Tissue damage can lead to increased concentrations of labile iron (Fe) in the interstitial fluid. Amyloid β can bind labile iron and undergo clearance via the glymphatics, transport across the blood–brain barrier (BBB), uptake by microglia and astrocytes, etc. With less available labile iron in the interstitial fluid, infections and redox-catalyzed tissue injury are reduced.

The transit of iron and other metals within the extracellular milieu can occur via carrier proteins, such as transferrin and ferritin [111,112], but during normal and pathological events (such as the breakdown of iron-containing proteins, aging, and inflammation), iron can also become independent of these proteins in the interstitial fluid [113,114,115,116,117] (Figure 1). In fact, non-transferrin-bound iron is normally present within extracellular fluids, such as the plasma and cerebrospinal fluid, as well as the interstitial fluid [114,118,119,120]. Inflammation (e.g., due to LPS) and amyloid β both increase the uptake of non-transferrin-bound iron by immortalized microglia, which is thought to limit the availability of extracellular iron for microbes [121]. During inflammation, nitric oxide levels are increased, due to the activation of inducible nitric oxide synthase [122], and iron can be liberated from ferritin by nitric oxide [123].

Leakage of the blood–brain barrier can also occur in response to inflammation, aging, and disease [61,124,125]. The extravasation of red blood cells can result in hemolytic processes and release of substantial amounts of hemoglobin and heme [126]. Heme and other hemoproteins are also released extracellularly following tissue damage [126,127]. Their removal and breakdown involve additional proteins.

Hemoglobin and heme will bind the plasma proteins haptoglobin and hemopexin, respectively. The hemoglobin-haptoglobin complex binds the CD163 transporter on macrophages/monocytes and is endocytosed and processed through the lysosome, which frees heme [126]. The heme-hemopexin complex is taken up by the LRP receptor, which is present on numerous cell types, and it undergoes endocytosis and lysosomal degradation resulting in heme release. Heme from these complexes, and heme from other hemoproteins that underwent lysosomal degradation, is delivered to the cytoplasm and is then broken down by heme oxygenase to biliverdin, carbon monoxide, and iron [126]. Besides being constitutively expressed (i.e., heme oxygenase-2), there can be induced expression (i.e., heme oxygenase-1), e.g., during stress, inflammation, free heme, and during aging within microglia [126,128,129]. The liberated iron from this reaction might facilitate tissue damage [128]. Furthermore, extracellular heme oxygenase 1 has been detected and may be a marker of disease activity [130].

Besides heme, iron is also thought to be released into the extracellular space following neurodegeneration, damage to the blood–brain barrier, demyelination, macrophages undergoing apoptosis, via glial cells, etc. [61,125,131,132,133]. In addition to moving iron from the lysosome to the cytosol, divalent metal transporter 1 (DMT-1) can take up ferrous iron from the extracellular space [126]. Ferric iron is first reduced by duodenal cytochrome B (DCYTB) to ferrous iron before internalization via DMT-1 [126,134,135].

If the liberated iron is not taken up by DMT-1, then it can associate with various ligands, but it would still be accessible for capture by microbial siderophores, which can then be taken up for utilization by these organisms [15,136,137]. Besides supporting infections [138,139,140], iron, particularly, non-transferrin bound or loosely bound iron (i.e., complexed to ligands or with other atoms), can partake in damaging chemical reactions, e.g., catalyzing the formation of reactive chemical species [13,14,15,16,17]. However, due to the ongoing production of amyloid β and its ability to bind iron and heme, it can likely serve a surveillance function to capture and remove this loosely bound iron (or ‘labile iron’). Thus, amyloid β could function as a mammalian siderophore, which has been postulated for other molecules in mammals that restrict iron from bacteria [141,142]. Amyloid β may thus carry out important protective functions to restrict iron availability for microbes and prevent iron from performing damaging chemical reactions (Figure 1); it may also participate in the recycling of iron, which could then be stored or reutilized in other biochemical functions.

## 7. Removal of Labile Iron from the Extracellular Milieu

Neurons have high nutrient requirements, and unlike other organs, they have limited ability to undergo replacement in the CNS following injury or infection. Thus, the blood–brain barrier of the CNS is critical to controlling the distribution of molecules into and out of the brain. This includes importing nutrients, restricting the entry of toxins, viruses, and bacteria, and eliminating waste products [143]. It is intuitive that the brain would utilize multiple measures for protection. Although the removal of amyloid β has been proposed to protect the brain from the toxic properties of this peptide, the clearance of amyloid β could serve other functions, such as removing or redistributing loosely bound iron from the interstitial fluid. The clearance of iron from the brain via amyloid β likely has the following multiple functions: (1) it limits iron availability for microbes; (2) it prevents against iron-catalyzed reactions that can cause brain tissue damage; and (3) it delivers or redistributes labile iron to other cells within and outside (e.g., liver) the CNS where it can be recycled for reuse or put into storage, e.g., bound to ferritin.

Amyloid β shares structural similarities with other antimicrobial peptides [144] and it has been proposed to function as part of the innate immune response to protect against infection [145,146,147]. There are many hypotheses for the antimicrobial activities of amyloid β including the following: it forms a pore that disrupts membranes; it entraps pathogens, thereby preventing their spread; it interferes with the adhesion of the pathogen to cellular surface proteins; it activates the immune response; etc. [148,149,150]. Some proteins, such as lactoferrin, can have dual antimicrobial functions by scavenging iron, thereby limiting its availability for microbes, as well as being cleaved into peptides with antimicrobial activity [151,152]. APP and amyloid β may share some properties with lactoferrin given the regulatory role on iron homeostasis by APP and the antimicrobial activity by amyloid β [153]. APP works with ferroportin to facilitate the export of iron from cells [43,47], but the excess labile iron would still need to be moved out of the CNS. Here, the notion is put forward that amyloid β binds loosely bound iron and exports it from the brain or redistributes it to other cells. This function would limit the availability of labile iron for microbes in the CNS.

Besides iron, amyloid β can bind other redox-active metals such as copper [154,155] and its removal could help limit damaging chemical reactions. Because copper is considered to be toxic to bacteria, its removal by amyloid β would not necessarily help eliminate microbes [156], though it would protect the brain from oxidative tissue damage. On the other hand, given that amyloid β can interact with microbes, it could deliver copper to microbes to promote their death by redox-active mechanisms. This would be consistent with the observation that copper is redox-active when bound to amyloid β [74,157,158] and that amyloid β interacts with microbes [145]. Whether iron is redox-active when bound to amyloid β is unclear [74,158,159,160], but it may be dependent on its concentration or whether amyloid β is in a soluble or aggregated form [73,161,162].

Redox-active metals, such as iron and copper (which are often associated with other molecules such as carboxylates, phosphates, and heme), can catalyze reactions that form highly reactive chemical species, which can then damage a range of biomolecules [13,14,15,16,17,75,76,163,164,165]. The binding of redox-active metals such as iron and copper to monomeric, but not oligomeric, amyloid β has been proposed to quench their ability to undergo reduction and thereby protect neurons from metal-catalyzed reactions [162]. A different study found that pretreatment for 24 h with secreted forms of APP protected cultured hippocampal neurons against iron-induced death while pretreatment with amyloid β exacerbated neuronal death [166]. The variance between results from these two studies [162,166] is likely due to whether amyloid β was in a monomeric form vs. being in an aggregated or oligomeric form, which could form during the 24 h pretreatment window. In fact, oligomeric amyloid β may contribute to oxidative damage, while monomeric amyloid β has antioxidant properties [162]. Regardless of whether metals are redox-active when bound to amyloid β, its clearance from the brain (or redistribution to other cells) would remove these metals from the interstitial fluid, thereby preventing their damaging reactions.

Amyloid β in the systemic circulation can be bound to circulating LRPs [167,168] and is thought to be cleared by the liver through endocytosis and lysosomal degradation or transcytosis (e.g., across hepatocytes with biliary excretion and the assistance of P-glycoprotein) [169,170]. In aged mice and rats, the uptake of amyloid β by the liver decreases and corresponds with decreased LRP-1 levels [171,172]. Circulating amyloid β may also be bound to ApoE, particularly ε2 or ε3, and undergo peripheral clearance [173,174], and LRP-1 facilitates the metabolism of ApoE containing lipoproteins [175]. LRP-1 also participates in iron regulation by internalizing heme/hemopexin complexes [176], and there is indirect evidence from ApoE- and hemopexin-deficient mice suggesting that LRP-1 is linked to hepatic lipid metabolism and iron hemostasis [177].

The liver is the major organ for iron storage and for regulating iron distribution in the body [178]. If iron was bound to amyloid β, the clearance of amyloid β by the liver would lead to the delivery and processing of iron by this organ for its recycled use or elimination. For example, iron can be utilized by liver cells to support their biochemical functions, put into storage in ferritin, or exported by ferroportin (whose function is regulated by hepcidin which is produced by the liver) [179], and after export associate with transferrin (which is also produced by the liver) [180]. Alternatively, after processing by the liver, iron could enter bile and be eliminated via feces or undergo reuptake by the small intestine [181].

## 8. Evidence Supporting That Amyloid β Functions as a Mammalian Siderophore

There is ample evidence supporting the role of amyloid β in clearing iron from the brain (Table 1). Both ferrous and ferric iron bind amyloid β [64,65,66,67,72], which suggests that unlike other iron-binding proteins, such as transferrin, it does not require an oxidase. Iron, particularly ferric iron, has a very high affinity for amyloid β [72], which is a requirement for an iron transport protein and helps restrict iron availability from microbes. Multiple atoms of iron can bind amyloid β [160], making it more efficient than other much larger iron transport proteins, such as transferrin, which binds only two atoms of ferric iron. The small size of amyloid β allows it to sequester iron from sites not accessible by other larger proteins involved in iron transport.

An infection can increase amyloid β production [182,183,184] and amyloid β increases the uptake of labile, non-transferrin bound iron by immortalized microglia [121]. Proinflammatory cytokines can be produced in response to an infection, aging, or disease [185,186], and IL-1 and other proinflammatory cytokines (TNF-α, IFN-γ, etc.) stimulate the production of APP and its processing into soluble forms of APP and/or amyloid β [187,188,189,190]. This response to increasing amyloid β levels would provide greater iron-capture capacity during a time when restricting iron availability would serve to limit the spread of an infection [60] (Figure 1).

Rather than evolutionarily selecting for its elimination, vertebrates expend significant energy and utilize multiple mechanisms to clear amyloid β from the CNS. This suggests that amyloid β performs valuable functions and that these functions are worth the expenditure even though it can mediate pathology over time. Various mechanisms are used to clear amyloid β, such as transcytosis across the blood–brain barrier, uptake by microglia or astrocytes, proteolytic degradation, elimination via the glymphatic system, etc. [78]. If iron is bound to soluble amyloid β, then as it is cleared, the iron could be readily removed from the brain or redistributed to other cells.

There are additional findings that peripherally support the role for amyloid β in the clearance of iron. The presence of iron induces the expression of ApoE [98], which can bind amyloid β and assist with its removal [84,92,93]. This would facilitate the removal of labile iron if it was captured by amyloid β. Other circumstantial findings include that overexpression of the carboxyl-terminal fragment of APP, which includes amyloid β, lowers iron and copper levels in the mouse brain [63]. Lowering APP levels causes an accumulation of iron in cultured primary neurons or HEK293T cells [43]. Mice deficient in APP had increased levels of iron in the CNS [46,191], and mice with both a partial deficiency of APP (heterozygous for APP knockout) and the Huntington’s disease mutation also had increased CNS iron levels [44]. Short peptides, whose design was based on the metal-binding properties of amyloid β, decrease iron levels from SH-SY5Y cultured cells, CSF, and the brain [192,193].

Together, these data indicate that amyloid β could limit iron availability from microbes to prevent or restrict infection.

## 9. The Presence of Iron in Amyloid Deposits

Iron is present throughout amyloid plaques, from the dense core to diffuse areas [68]. Much of this iron is tightly bound, e.g., it can be uncovered after treatment of CNS tissue with proteinase K and/or detergents [68,70]. It is unlikely that plaques form first and then iron binds; rather, iron likely binds amyloid β before it aggregates. In fact, iron may act as a catalyst to promote the formation of fibrils and aggregation of amyloid β [66,194,195]. The uptake of amyloid β fibrils by microglia [79] may also remove any bound iron. If amyloid β is not cleared in a timely or efficient manner, for example, due to decreased expression of LRP-1 with aging or decreased clearance of ApoE4/amyloid β [92,93,196,197,198], then the iron can facilitate further aggregation of amyloid β and the formation of plaques. Besides amyloid β removing labile iron from the interstitial fluid, having iron tightly bound within the plaques could also be a mechanism to make iron less accessible for microbes.

Vessels and smooth muscle cells can take up amyloid β [90,96] and it can be synthesized and processed at the vessels [124,199,200]. Iron colocalizes with amyloid β and calcium in deep regions of perforating arteries in the cortex of patients with hereditary cerebral hemorrhage with amyloidosis, Dutch type, and sporadic cerebral amyloid angiopathy [201]. In Alzheimer’s disease, patients with cerebral amyloid angiopathy, which is a common occurrence in this disease [124], some large vessels and capillaries within the entorhinal cortex/hippocampus had iron deposition, but the extent of iron deposition is unknown, and detection may be dependent on the histochemical staining procedure employed [202].

The accumulation of iron in CNS vessels in Alzheimer’s disease and in cerebral angiopathy may result from iron getting ‘stuck’ during transit through vessels to amyloid β deposits, or from iron binding to amyloid β prior to its deposition in vessels. It is unclear if the deposited iron contributes to the impaired vessel function or the development of hemorrhagic lesions that occur in cerebral amyloid angiopathy [203]. It is possible that the iron is redox-reactive, which could cause tissue injury at and around vessels [13,14,15,16,17,204], although it is unclear if iron can act as a catalyst when bound to amyloid β [73,74], but heme appears to be redox-reactive after it binds amyloid β [75,76].

## 10. Remaining Work

We have discussed evidence that supports amyloid β functioning to remove labile iron from the interstitial fluid in the CNS, but several items still need to be demonstrated to support the validity of this mechanism (Table 1).

(1) During normal and disease conditions, does amyloid β in the interstitial fluid and CSF have iron or other redox-active metal bound to it? If amyloid β serves a surveillance function to capture labile iron, it is likely that amyloid β is in great excess relative to the loosely bound iron, especially since the distribution of amyloid β would cover the entire extracellular volume in the CNS. Furthermore, the amount of liberated iron or other redox-active metal is likely to be low, particularly during normal conditions. This is based on a couple of findings. Free or labile extracellular iron remains very low in plasma following the administration of a bolus of iron [120]. There are uptake mechanisms for non-transferrin-bound iron [205,206], which function in multiple cell types in the brain [115]. Thus, the percentage of amyloid β with bound iron would likely be under one percent during normal conditions, but would increase during pathological conditions, especially during infection, inflammation, seizure, or traumatic brain injury. Also, the number of iron atoms bound to each molecule of amyloid β would be expected to be low or zero during normal conditions and increase during pathological conditions where there could be the liberation of iron concentrated from one or more sites (Figure 1).

(2) Can amyloid β with a bound redox metal(s) be cleared from the brain? Can it cross the blood–brain barrier (e.g., via LRP-1), be taken up by microglia or astrocytes, or be removed by the glymphatic system? Careful consideration will need to be taken with designing experiments and interpreting results, particularly for in vitro studies. As mentioned previously, the percentage of amyloid β with iron bound to it in vivo would be expected to be very low and matching this in vitro could prove difficult. If the concentration of iron is too high, then this could cause fibril or aggregate formation, especially over time, which could interfere with the ability to clear amyloid β. In cell culture experiments that that study amyloid β uptake or that mimic the blood–brain barrier, iron is present in media, such as Dulbecco’s Modified Eagle’s Medium, and it is in relatively high concentrations in fetal bovine serum [207]. Thus, the concentration of labile iron in standard, in vitro conditions may be physiologically irrelevant.

(3) Does the absence of amyloid β increase the risk of infections or metal-catalyzed oxidative damage in the brain? Designing experiments that address these questions may be difficult since the brain has redundant functions to fight infections and limit tissue damage (e.g., antioxidants like glutathione). The brain also has redundant ways to capture and remove labile metals, such as albumin [131] and DMT-1 [126], respectively. Furthermore, amyloid β has antimicrobial properties; thus, besides a false negative result being due to a redundant function, a false positive result may be due to a missing mechanism unrelated to iron; thus, the data should be interpreted cautiously. For example, mice deficient in APP showed a trend to be more susceptible to infection by *Salmonella enterica* serotype Typhimurium [145], but it is unknown if more labile iron was available for infection or if the effect was due to the absence of amyloid β, which can have antibacterial effects independent of iron capture.

## 11. CNS Clearance of Amyloid β in Patients with Alzheimer’s Disease

The clearance of amyloid β from the CNS is decreased in patients with Alzheimer’s disease compared to control subjects. This may be related to the aggregation of amyloid β and its deposition into plaques [208,209], and studies suggest that elevated brain iron promotes the deposition of amyloid β [210]. In addition to aggregation, clearance mechanisms may also be impaired in Alzheimer’s disease, which can contribute to the reduced removal of amyloid β [211,212]. LRP levels in the midfrontal cortex were found to decrease with age, and this was not simply due to synaptic or neuronal loss, since this decrease was observed even when levels were normalized to the levels of synaptophysin besides actin [213]. Furthermore, when compared to actin levels, LRP levels were approximately two-fold lower in patients with Alzheimer’s disease compared to age-matched healthy control subjects, and higher LRP levels were associated with disease onset at a later age [213]. An inverse correlation was observed for vascular expression of P-glycoprotein and amyloid β plaques in the medial temporal lobe, suggesting that decreased activity in this transporter caused greater extracellular accumulation of amyloid β [214]. LRP-1 mRNA and protein levels were decreased with advanced aging in rats, and P-glycoprotein also had a biphasic response with a large decrease at an advanced age [197,198].

Although studies suggested that elevated brain iron promotes the deposition of amyloid β [210], an alternative explanation could be true—that the inability to clear amyloid β leads to its aggregation and the deposition of the iron to which it was bound, or will bind, and this contributes to the increase in brain iron levels in Alzheimer’s disease [215].

## 12. Is Anemia of Inflammation a Contributing Pathogenic Mechanism in Alzheimer’s Disease?

Iron is a critical nutrient for bacteria and other microbes. In response to invading microbes (or in response to other conditions such as cancer), the availability of iron is reduced to limit the infection or the chronic pathological process. Anemia of chronic disease (or anemia of inflammation) occurs when proinflammatory cytokines (e.g., IL-1, IL-6, TNF, and interferon) are increased in response to an ongoing condition. Proinflammatory cytokines stimulate the transcription of the hepcidin antimicrobial peptide (HAMP) gene that encodes for hepcidin [216]. Hepcidin also can be produced by macrophages and microglia [216,217]. Hepcidin then interacts with membrane-bound ferroportin, which causes its internalization and degradation, e.g., in cell cultures and monocytes/macrophages [58,216,218]. This in turn decreases iron export via ferroportin, resulting in reduced iron uptake by the gastrointestinal tract, lower levels of circulating iron, and iron retention, i.e., in macrophages [219,220,221].

Similar mechanisms are thought to act in the brain [222]. For instance, in response to inflammation (TNFα, IL-6, and LPS), mRNA and immunofluorescence for hepcidin were increased in cultured astrocytes and microglia, and hepcidin administration decreased ferroportin expression in these cells, as well as in neurons [223]. DMT-1 was also increased in all three cell types in response to inflammatory stimuli. These changes possibly accounted for the accumulation of iron in the neurons and microglia [223]. In Alzheimer’s disease, IL-6 is thought to induce the expression of hepcidin, which suppresses ferroportin on neuronal cells, resulting in the accumulation of iron [224]. Also, in response to inflammation or an infection, IL-1 is produced and increases the expression of APP, due to the IL-1 responsive acute box element in the 5′-UTR, which in turn can increase the production of amyloid β [27]. Increased amyloid β could reduce the amount of labile iron in the brain by facilitating its export from the CNS, thereby limiting an infection and reducing damaging metal-catalyzed reactions.

Studies indicate that anemia and anemia of inflammation are more common in patients with Alzheimer’s disease or other dementias [225,226]. Anemia of inflammation has been proposed to occur in multiple system atrophy where increases in iron in the pons, together with ferritin accumulation in reactive microglia, were accompanied by a decrease in ferroportin together with possible increases in hepcidin and IL-6 [227]. Similarly, a situation where iron accumulates in the CNS but is unavailable for use (a functional iron deficiency) has been proposed as a pathogenic mechanism for Alzheimer’s disease [228,229]. Besides iron retention due to lowered ferroportin levels, as a result of events similar to anemia of chronic disease, iron can also become unavailable for use by other mechanisms, e.g., sequestered by amyloid β, plaques, tau, impaired lysosomes, and reactive microglia [229,230]. Together, these responses would interfere with the export of iron from the brain by amyloid β.

## 13. Conclusions

We have discussed evidence supporting that amyloid β, in its soluble form, functions to clear labile iron from the brain (Table 1). The evidence suggests that this clearance is performed in an efficient manner as follows: due to its small size, amyloid β can access sites that larger proteins cannot; it can bind to multiple atoms of both ferrous and/or ferric iron without an oxidase; it is produced recurrently, allowing it to perform a surveillance function; it has feedback mechanisms to increase its production in the presence of excess iron, inflammation, or infection; and it is cleared from the brain by multiple redundant mechanisms. This function does not replace the role for the clearance of amyloid β to prevent its aggregation in the brain but would instead complement it. Further investigation is still necessary to establish that a role of amyloid β clearance is the export of labile iron from the interstitial fluid of the CNS (Table 1).

By removing labile iron from the extracellular space, amyloid β keeps iron away from invading microorganisms, thereby preventing or limiting an infection. Furthermore, the clearance of iron-bound amyloid β would deliver the iron to other cells for reuse or storage. For example, iron-bound amyloid β can be taken up by microglia, astrocytes, or transported out of the brain and be recycled by the liver. Therefore, amyloid β is essentially acting as a mammalian siderophore. In addition, amyloid β is preventing redox chemical reactions from causing tissue damage by removing loosely bound iron in the interstitial fluid.

If the removal of extraneous iron by amyloid β prevents tissue injury and infection, would disrupting this process contribute to disease? During aging, the clearance of amyloid β from the extracellular fluid can decrease, e.g., due to lower expression of LRP-1 receptor [197,198]. Less clearance allows greater opportunity for amyloid β to form fibrils and aggregate and iron may facilitate this process. The iron bound to amyloid β in plaques and vessels would be less available to microbes than labile iron, but since some bacteria and their products have been found to be present in plaques [231,232,233,234,235], it is possible that the iron can leach out over time and support microbial growth. Similarly, iron bound to plaques and vessels likely causes less tissue damage than labile iron, but whether this iron is redox-active is uncertain [74] and it is possible that iron deposited with amyloid β at vessels promotes additional pathology, e.g., hemorrhagic lesions, and impairs vessel function [203].

When iron is captured in extracellular aggregates of amyloid β, less iron would be recycled for reuse. In Alzheimer’s disease, additional factors contribute to the generation of a functional iron-deficient state [228,229]. For example, the lysosome can have impaired processing of iron for recycling (e.g., diminished mitophagy or ferritinophagy) or decreased delivery of iron to the cytosol, iron can become bound by tau in addition to amyloid β, iron can become trapped in microglia, etc. [215,230]. Due to the ongoing inflammation, there is also likely anemia of chronic disease occurring in Alzheimer’s disease, which involves elevated production of hepcidin and reduced expression of ferroportin, resulting in less available iron [224]. This could compound the effects from the iron that is sequestered during disease, making less iron available for use. A functional deficiency of iron can impair multiple processes within neurons, oligodendrocytes, and other cells in the brain [229,230]. Numerous iron- or heme-containing proteins are susceptible to a deficiency of iron, including mitochondrial complexes I and IV [236,237,238], and impaired complex and mitochondrial function has been observed in Alzheimer’s disease [229,239].

The clearance of iron and other redox-active metals by amyloid β may be a critical function to help preserve brain health by protecting it from redox-mediated tissue damage and the development and spread of infections. Given that oral bacteria and other infections that enter the blood stream have the potential to access the brain [240,241], the clearance of labile iron by amyloid β would have been particularly valuable to our ancestors when oral hygiene was comparatively poor and antibiotics were not available. The importance of the clearance of iron by amyloid β within the brain may have been evolutionarily selected for, even at the expense of managing a peptide with the potential to mediate pathology.

## Figures and Tables

**Table 1:** **Amyloid β clearance of labile iron from the CNS** ^1^.

Supporting Evidence ^2^	Comment
Amyloid β binds both ferrous and ferric iron	An oxidase is not required for binding
Amyloid β has a relatively high affinity for iron	Would keep iron bound to it during export and may limit iron-catalyzed redox reactions
Amyloid β is regularly produced from multiple sites and enters the interstitial fluid	Provides a surveillance function to rapidly capture labile iron
Amyloid β is cleared by a variety of mechanisms	Ensures that labile iron is removed from the interstitial fluid and facilitates the delivery of iron for reuse by other cells
Amyloid β is relatively small	Enables it to access sites unavailable to larger proteins
Amyloid β can bind multiple atoms of iron	Can transport more iron than some other iron transport proteins. This could be advantageous during infections or disease, when there could be localized areas of relatively high concentrations of liberated iron
Amyloid β expression can increase during infection, inflammation, or high iron levels	This response can reduce the availability of labile iron during times of need, i.e., when it would be helpful to have less available iron
Amyloid β is conserved among vertebrates	The benefits of amyloid β outweigh its negative potential for neuropathology
**Work Remaining**	**Comment**
Demonstrate that soluble amyloid β in the interstitial fluid has bound iron	Only a very small fraction of amyloid β would be expected to have iron bound during normal states, but this fraction may increase when the liberation of iron is increased, e.g., during tissue injury
Demonstrate that amyloid β with bound iron can undergo clearance	For in vitro studies, iron and amyloid β concentrations and time in culture can affect amyloid β aggregation, which can influence transport
Demonstrate that failing to clear labile iron causes increased tissue damage and risk of infections	Experimental conditions may not reflect natural circumstances, or redundant protective mechanisms could counter or mask effects

^1^ see text for references. ^2^ except for the final item in this top section, the listed properties suggest that amyloid β would clear labile iron from the CNS in an efficient manner.

## Data Availability

No new data were created or analyzed in this study. Data sharing is not applicable to this article.
